# Complete mitochondrial genomes of *Culicoides brevitarsis* and *Culicoides imicola* biting midge vectors of Bluetongue Virus

**DOI:** 10.1080/23802359.2024.2447750

**Published:** 2025-01-03

**Authors:** Khandaker Asif Ahmed, Anjana Karawita, Melissa J. Klein, Luana Fiorella Mincarelli, Barbara Secondini, Giuseppe Satta, Massimo Ancora, Cipriano Foxi, Marco Di Domenico, Michela Quaglia, Maria Goffredo, Alessio Lorusso, Cesare Cammà, Leon Court, Rahul V. Rane, Tom K. Walsh, Prasad N. Paradkar, Debbie Eagles, Gunjan Pandey, Christopher M. Hardy

**Affiliations:** a CSIRO Australian Animal Health Laboratory (AAHL), Australian Centre for Disease Preparedness (ACDP), East Geelong, VIC, Australia; b CSIRO Health and Biosecurity (H&B), Australian Centre for Disease Preparedness (ACDP), East Geelong, VIC, Australia; c National Reference Centre for Whole Genome Sequencing of microbial pathogens – Istituto Zooprofilattico Sperimentale dell’Abruzzo e del Molise, Teramo, Italy; d Istituto Zooprofilattico Sperimentale Della Sardegna, Sassari, Italy; e CSIRO Environment, Black Mountain, ACT, Australia; f CSIRO Health and Biosecurity (H&B), Parkville, VIC, Australia

**Keywords:** Arbovirus vector, *Culicoides*, Ceratopogonidae, biting midges, mitogenome

## Abstract

Biting midges (*Culicoides* spp.) are important vectors of several insect borne arboviruses but are underrepresented in terms of availability of high-resolution genomic resources. We assembled and annotated complete mitochondrial genomes for two *Culicoides* species, namely *C. brevitarsis* and *C. imicola* which are proven vectors for Bluetongue Virus (BTV). We used both long read and short read sequencing technologies to assemble the circular genomes. The genome sizes are 17,100 bp and 17,031 bp, respectively, all comprising 37 genes, including 13 protein, 22 tRNA, two rRNA coding genes, and one non-coding AT rich control region. The gene organizations and orientations are comparable to other available *Culicoides* mitogenomes, except for a translocation of *ND2* and six tRNA genes in both *C. brevitarsis* and *C. imicola*. Eleven protein-coding genes encode a full TAA stop codon, while two (*ND5*, *COX3*) are completed by mRNA polyadenylation. Phylogenetic analysis of the mitogenomes showed *C. brevitarsis* and *C. imicola* form a monophyletic group. The sequences of these mitogenomes contribute to a baseline of molecular tools for diagnostics and surveillance for use by World Organisation for Animal Health (WOAH) reference laboratories for monitoring vectors of emerging diseases.

## Introduction

Biting midges (*Culicoides* spp.) are the largest genus in the Ceratopogonidae family, comprising >1300 species worldwide (Bellis et al. 2007). Among different species, the African and middle eastern species *Culicoides imicola* (Kieffer, 1913), Australasian species, *Culicoides brevitarsis* (Kieffer, 1917), and North American, *Culicoides sonorensis* (Wirth & Jones, 1957) are all significant for transmitting several arboviruses of agricultural and veterinary importance, including Bluetongue Virus (BTV), Epizootic Hemorrhagic Disease Virus, African Horse Sickness Virus, Akabane Virus, and Lumpy Skin Disease Virus (Bellis et al. 2007; Mellor et al. 2009; Kampen et al. 2023; Quaglia et al. 2023). Even though there are numerous surveillance and vector competency studies on these species, there is a lack of high-quality genomic resources, which can be crucial for genomic engineering and genetic mitigation programs. As a part of World Organisation for Animal Health (WOAH) reference laboratories for BTV in Australia and Italy, we are developing genomic resources to support all reference laboratories and researchers working on *Culicoides*. Here, we describe complete, high quality reference mitochondrial genomes for two *Culicoides* species and an analysis of mitogenome structure compared to other biting midges (*Forcipomyia* and *Culicoides*).

## Methods

**Figure 1.**
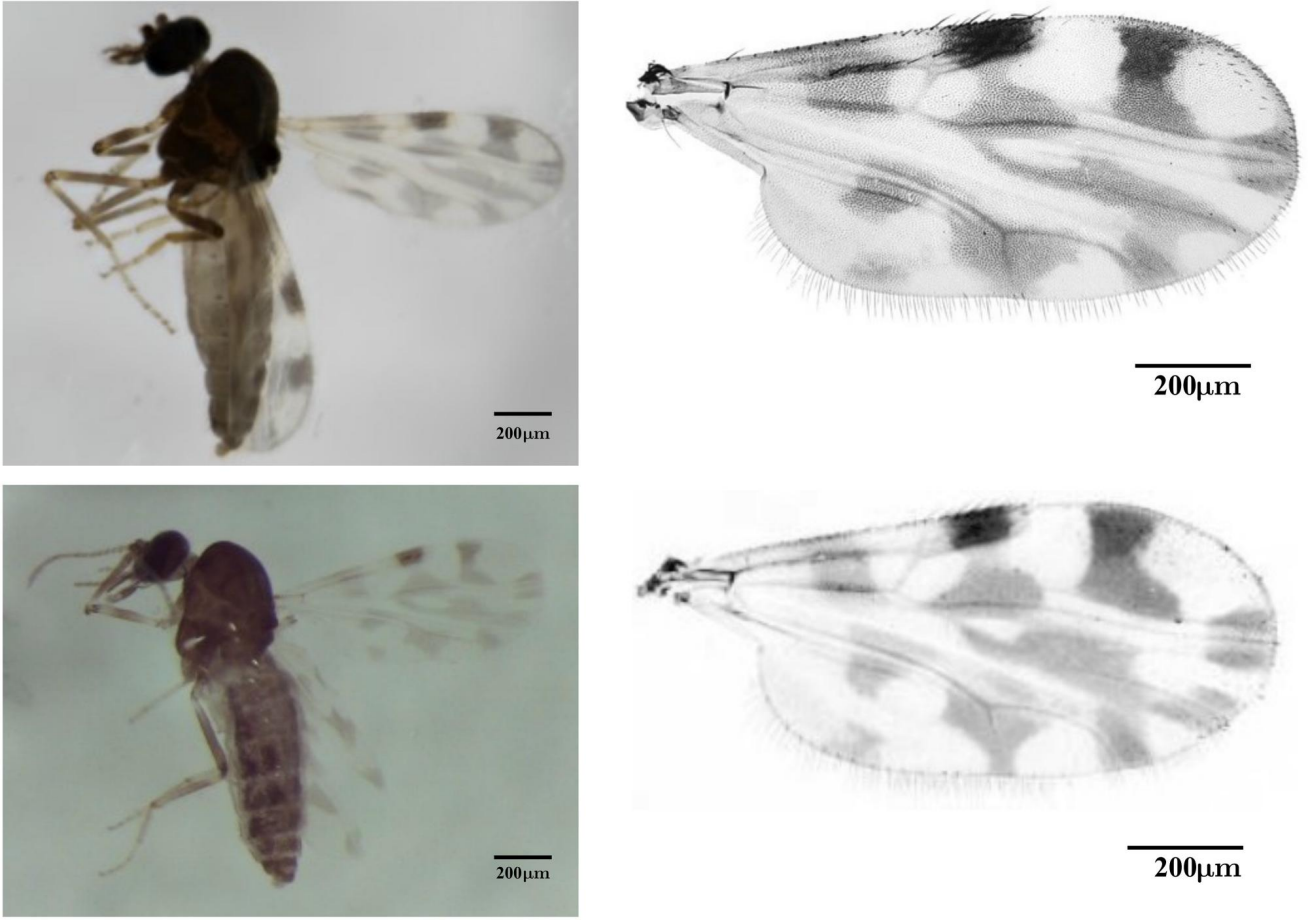
Whole body images and wing venation patterns of two *Culicoides* species reported in current study. Top and bottom panel show *C. brevitarsis* and *C. imicola*, respectively. Whole body images are taken by the authors. Wing venation slide preparations and pictures were taken by Dr. Glenn Bellis (unpublished, original work) with the author’s written permission to use.

*C. brevitarsis* was collected from a cattle farm at Casino, NSW, Australia (28°52′0″S 153°03′0″E) and morphologically identified based on wing patterns and venation (Bellis et al. 2007) (Figure 1). Total DNA was extracted using a Blood & Cell Culture DNA Mini Kit (Cat: 13323, Qiagen, Hilden, Germany) from a pool of 50 females. A ligation based genomic DNA library was prepared using a ligation sequencing kit (Cat: SQK-LSK114, Oxford Nanopore, Oxford, UK) and sequenced in a PromethION flowcell (Cat: FLO-PRO114M, Oxford Nanopore, Oxford, UK). For *C. brevitarsis*, total RNA was extracted, using Qiagen RNeasy Plus Mini Kit (Qiagen, Germantown, MD), in three replicates, each containing 10 whole individuals. Subsequent library preparation and short-read based sequencing (150 bp pair-end reads with over 50 million reads per replicate) were commercially outsourced to Azenta (Beijing, China).

*C. imicola* was collected from a sheep farm at Piscinas (Sardinia), Italy (39°04′26″N 8°39′58″E) and morphologically identified (Figure 1) (Delécolle 1985). Total DNA was extracted from an individual insect using the High Pure Viral Nucleic Acid Kit (Cat: 11858874001, Roche, Basel, Switzerland), including an incubation with Proteinase K at 56 °C for 2 h. Samples were sequenced with the Nextera DNA Flex Illumina sequencing protocol for low-input DNA samples (12 cycles BLT PCR) on an Illumina P2 NextSeq2000 cartridge (San Diego, CA) returning 2 × 150 bp paired reads.

*C. sonorensis* mitogenomes have previously been reported (Milián-García et al. 2022) but an annotated version was not available in GenBank. Therefore, we used Illumina raw reads available in GenBank for a pool of 100 females from a colony of *C. sonorensis* (SONORA strain) (SRA Accession: ERR1879555; Bioproject: PRJEB19938) to conduct a third party mitogenome assembly and annotation (BK065013). These data were derived from specimens collected from a human sewage effluent site, at Sonora, Texas (30°34′5″N 100°38′39″W) and a cell line and previously used to create a nuclear genome assembly (GCA_900002565) (Morales-Hojas et al. 2018). This assembly is also reported to contain a mitochondrial scaffold (Genome scaffold 710: LN484060, unverified mitochondrial genome accession: ON758298) for this species (Milián-García et al. 2022). RNASeq data available in GenBank from a cell line were used for annotation of stop codons (SRR26081355; Bioproject PRJNA1018134) (McHolland and Mecham 2003; Scroggs et al. 2023). Specimens of *C. brevitarsis* and *C. imicola* are deposited at ACDP Geelong and IZS Teramo, respectively (Contact: Asif Ahmed, Khandakerasif.ahmed@csiro.au or Alessio Lorusso, a.lorusso@izs.it) under the voucher numbers Cbre2023 and Cimi001.

Data QC and quality trimming, and assembly of contiguous unambiguous consensus sequences were conducted using CLC Genomics Workbench 23.0.4 (Qiagen, Germantown, MD). Assemblies were confirmed visually by mapping raw reads back to each assembly (Figure S1). Mitogenome annotations were identified in Geneious Prime 2020.1.2 (Biomatters Ltd, Auckland, New Zealand) using the *C. arakawae* and *C. stellifer* mitogenomes AB361004 and PP873183 as references. All available Ceratopogonidae mitochondrial assemblies in NCBI and any other mitogenome that shared greater than 80% nucleotide identity by BLASTn to those already identified were included for phylogenetic analyses. *Aedes aegypti* (Diptera, Culicomorpha, Culicidae) was chosen as a reliable outgroup. Four species of chironomids (Diptera, Chironomidae, *Chironomus*) were also added in the phylogenetic tree based on a previous phylogenomic studies placing *Culicoides* within Culicomorpha (Kutty et al. 2018). The 13 mitochondrial protein genes for each species were then extracted and aligned individually using MAFFT v7.2.1.5 (Katoh and Standley 2013). Aligned sequences were then concatenated for each species, and optimal model selection, ML-based phylogeny and bootstrap analysis were conducted using IQ-Tree with 1000 bootstrap replicates (Nguyen et al. 2015). The consensus tree was visualized using iTol web server v. 6.9.1 (Letunic and Bork 2024).

## Results

**Figure 2.**
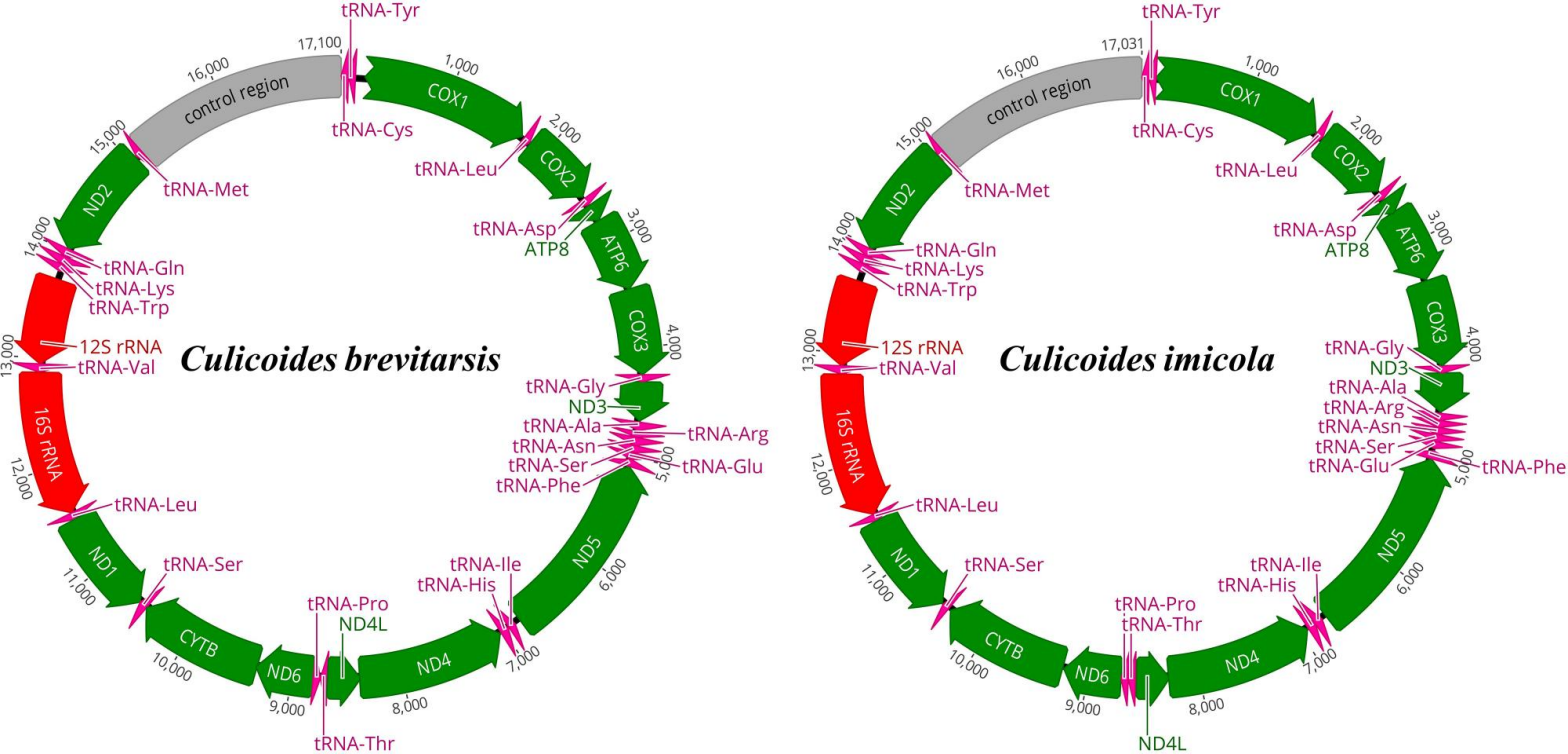
Circular mitochondrial genome feature plots of two *Culicoides* species reported in current study. Green, red, pink, and grey colored bars showing protein coding, rRNA, tRNA, and CR genes, respectively. Arrows indicate the orientations of the specific genes.

The mitogenomes of *C. brevitarsis* and *C. imicola* were similar in length (17,100 bp and 17,031 bp, respectively), but slightly longer compared to *C. sonorensis* (16,060 bp). The sequence of our third-party *C. sonorensis* assembly is 653 nt longer than the 15,407 bp previously reported (ON758298) due to our detection and inclusion of additional direct repeat units within the CR into our assembly. The mitogenome size differences between the three species are mainly attributable to differences in the length of the control region (CR) (Figure 2). The longer CRs of *C. brevitarsis* and *C. imicola* both contain direct and inverted repetitive sequences separated by dinucleotide AT repeat sequences of 258 bp (nts 16,218–16,791) and 102 bp (nts 16,446–16,547), respectively. These AT repeats occur at sites in the mitogenomes where we observe a greater than 20-fold reduction in sequence mapping coverage for Illumina (from 48,664× to 2517× for *C. imicola*) as well as PromethION (from 554× to 10× for *C. brevitarsis*) reads (Figure S1). Read coverage reduction is not observed for the *C. sonoriensis* mitogenome CR which contains only a direct repetitive sequence element.

All three mitogenomes contain 37 genes, comprising 13 protein-coding genes (PCGs), two rRNA, 22 tRNA genes, and an AT rich non-coding CR (Figure 2). The number, order, and orientation of genes in *C. brevitarsis* and *C. imicola* are identical but differ to *C. sonoriensis* and other species of Ceratopogonidae biting midges (*C. arakawae*, *C. stellifer*, *Forcipomyia makanensis*, and *F. pulchrithorax*) by the translocation of six tRNA genes and one PCG (Figure S2). Six PCGs, *COX1*, *COX2*, *ATP8*, *ATP6*, *COX3*, *ND3*, *ND6*, and *CYTB* are in plus frame, and 5 (*ND5*, *ND4*, *ND4L*, and *ND1*) are in minus frame in all species (Figure 2). The only PCG order difference involves the *ND2* gene. This gene is in the minus frame between the 12S rRNA and CR in both *C. brevitarsis* and *C. imicola* but is in plus frame before *COX1* in *C. sonorensis* and other *Culicoides* and *Forcipomyia* species. The 12S rRNA and 16S rRNA genes were in the minus frame in all mitogenomes (Figure 2). The six translocated tRNA genes in *C. brevitarsis* and *C. imicola* encode tRNA-Gln, tRNA-Ile, tRNA-Lys, tRNA-Met, tRNA-Ser, and tRNA-Trp. The TAA stop codon for two genes (*ND5* and *COX3*) appears to be completed by the addition of adenine residues to the mRNAs. This was confirmed for both genes in *C. brevitarsis and C. sonorensis* by mapping RNAseq data available in GenBank (SRR27870270 and SRR26081355, respectively) back to the mitogenomes. The start codon for *COX1* was not determined in all three species.

**Figure 3.**
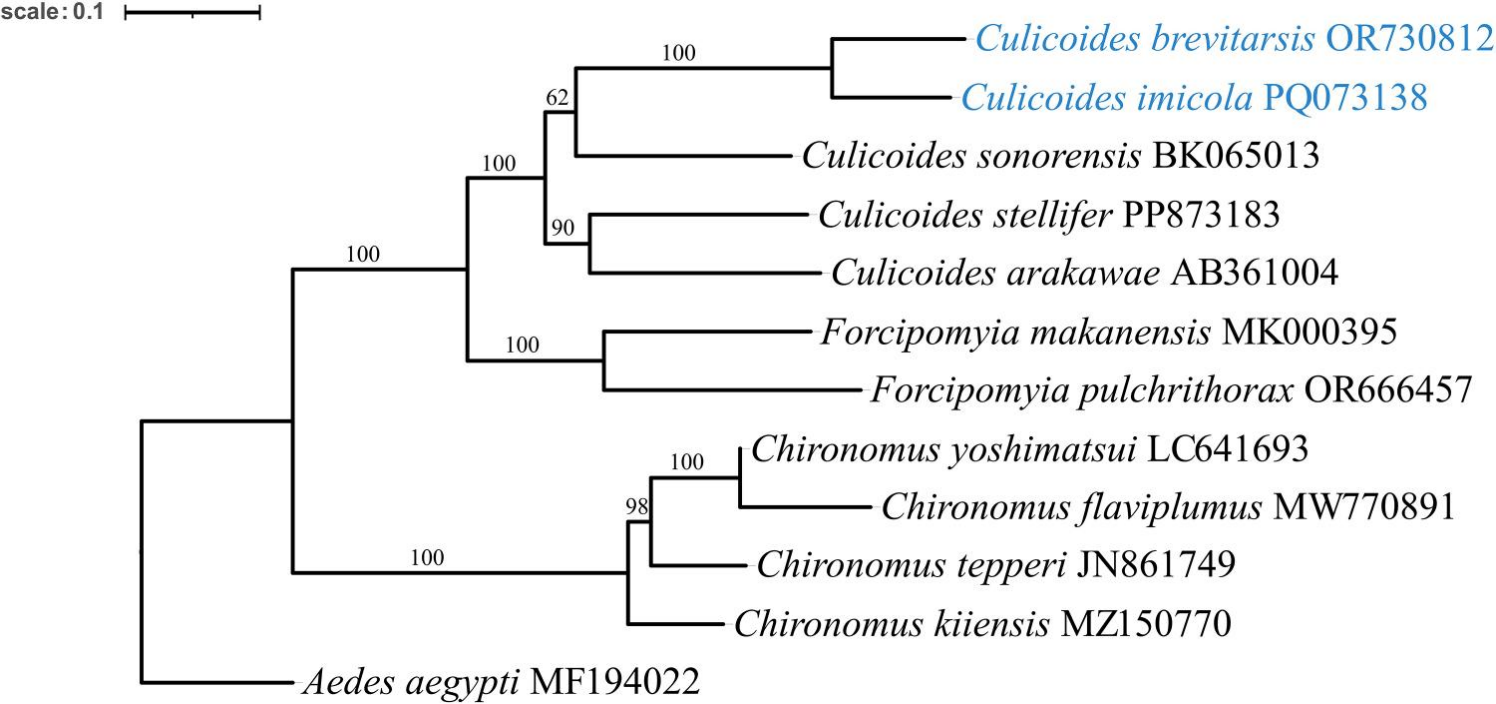
Maximum-likelihood phylogenetic tree, including bootstrap values, showing different biting and nonbiting midge mitogenomes. The mitogenomes presented in the current study are shown in blue. *Aedes aegypti* (MF194022) has been used as outgroup. The 13 individually aligned mitochondrial protein coding regions were ligated in tandem for each species in the analysis. The following source mitogenomes were used: *C. brevitarsis* (OR730812) (current study), *C. imicola* (PQ073138) (current study), *C. sonorensis* (BK065013) (Milián-García et al. 2022, current study), *C. arakawae* (AB361004) (Matsumoto et al. 2009), *C. stellifer* (PP873183) (Castellanos-Labarcena et al. 2024), *F. makanensis* (MK000395) (Jiang et al. 2019) and *F. pulchrithorax* (OR666457) (Karademir et al. 2023), *Chironomus tepperi* (JN861749) (Beckenbach 2012), *Chironomus yoshimatsui* (LC641693) (Hiki et al. 2021), *Chironomus flaviplumus* (MW770891) (Park et al. 2021), and *Chironomus kiiensis* (MZ150770) (Liu et al. 2022). The scale bar shows substitutions per site. One thousand bootstrap replicates were used.

A maximum-likelihood tree (Figure 3) confirms *C. brevitarsis* and *C. imicola* to be the most closely related phylogenetically, and they form a monophyletic group with the *C. sonorensis*, sister to the other two *Culicoides* species (*C. stellifer* and *C. arakawae*). It is also evident that species of *Chironomus* genera are more distantly related to *Culicoides*, compared to *Forcipomyia* genera (*F. makanensis* and *F. pulchrithorax*).

## Discussion and conclusions

This study presents the mitogenome assemblies of two biting midges and compares their structural variation with other biting midges. The genome organization is comparable to other midge mitogenomes, as well as an outgroup mosquito mitogenome, apart from a change in the location of the *ND2* gene and six tRNAs (tRNA-Gln, tRNA-Ile, tRNA-Lys, tRNA-Met, tRNA-Ser, and tRNA-Trp) in *C. brevitarsis* and *C. imicola*. Phylogenetic analysis highlights a close relationship between *C. brevitarsis* and *C. imicola*. Our study provides baseline resources to study taxonomy, phylogeny and evolutionary relationship across different *Culicoides* species and develop novel biomarkers for host specificity, diagnostics, and surveillance studies.

## Supplementary Material

supplementary_figs.pdf

## Data Availability

The genome sequence data that support the findings of this study are openly available in GenBank of NCBI at https://www.ncbi.nlm.nih.gov/ under the accession numbers OR730812, PQ073138, and BK065013. The associated BioProject, SRA, and Bio-Sample numbers are PRJNA1059659, SRR31254258, SRR27870270, SAMN39213272 and PRJNA1155864, SRR31654853, SAMN43472358 *C. brevitarsis*, and *C. imicola*, respectively.
