# Supplementary material for: Complete mitochondrial genomes of Culicoides brevitarsis and Culicoides imicola biting midge vectors of Bluetongue Virus
Source: Mitochondrial DNA B Resour. 2025 Jan 3;10(1):67–71. doi: 10.1080/23802359.2024.2447750 (PMC11703489; doi:10.1080/23802359.2024.2447750)

Complete mitochondrial genomes of *Culicoides brevitarsis* and *Culicoides imicola* biting midge vectors of Bluetongue Virus

Supplementary materials

Khandaker Asif Ahmed[1*], Anjana Karawita[1], Melissa J Klein[2], Luana Fiorella Mincarelli[3], Barbara Secondini[3], Giuseppe Satta[4], Massimo Ancora[3], Cipriano Foxi[4], Marco Di Domenico[3], Michela Quaglia[3], Maria Goffredo[3], Alessio Lorusso[3], Cesare Cammà[3], Leon Court[5], Rahul V. Rane[5], Tom K Walsh[5], Prasad N Paradkar[2], Debbie Eagles[1], Gunjan Pandey[5], Christopher M. Hardy[5]

[1]CSIRO Australian Animal Health Laboratory (AAHL), Australian Centre for Disease Preparedness (ACDP), East Geelong, VIC 3220, Australia.
[2]CSIRO Health and Biosecurity (H&B), Australian Centre for Disease Preparedness (ACDP), East Geelong, VIC 3220, Australia.
[3]National Reference Centre for Whole Genome Sequencing of microbial pathogens - Istituto Zooprofilattico Sperimentale dell'Abruzzo e del Molise, 64100, Teramo, Italy
[4]Istituto Zooprofilattico Sperimentale Della Sardegna, 07100, Sassari, Italy.
[5]CSIRO Environment, Black Mountain, ACT 2601, Australia.

*Corresponding author: khandakerasif.ahmed@csiro.au

Supplementary Figure S1: Read coverage plots for mitogenome assemblies of *Culicoides brevitarsis* (10-554X PromethIon coverage), *C. imicola* (2,517-48,664X Illumina coverage) and *C. sonorensis* (539-6,155X Illumina coverage)**.** Reads were mapped back to each assembled linearised mitogenome using CLC Genomics 23.0.4 with stringency settings of 0.80 read length and 0.99 nucleotide identity. Unpaired forward (green), reverse (red) and paired (blue) are shown. Reads able to be mapped to more than one location in the Control Region due to the presence of repeat sequences are in yellow.

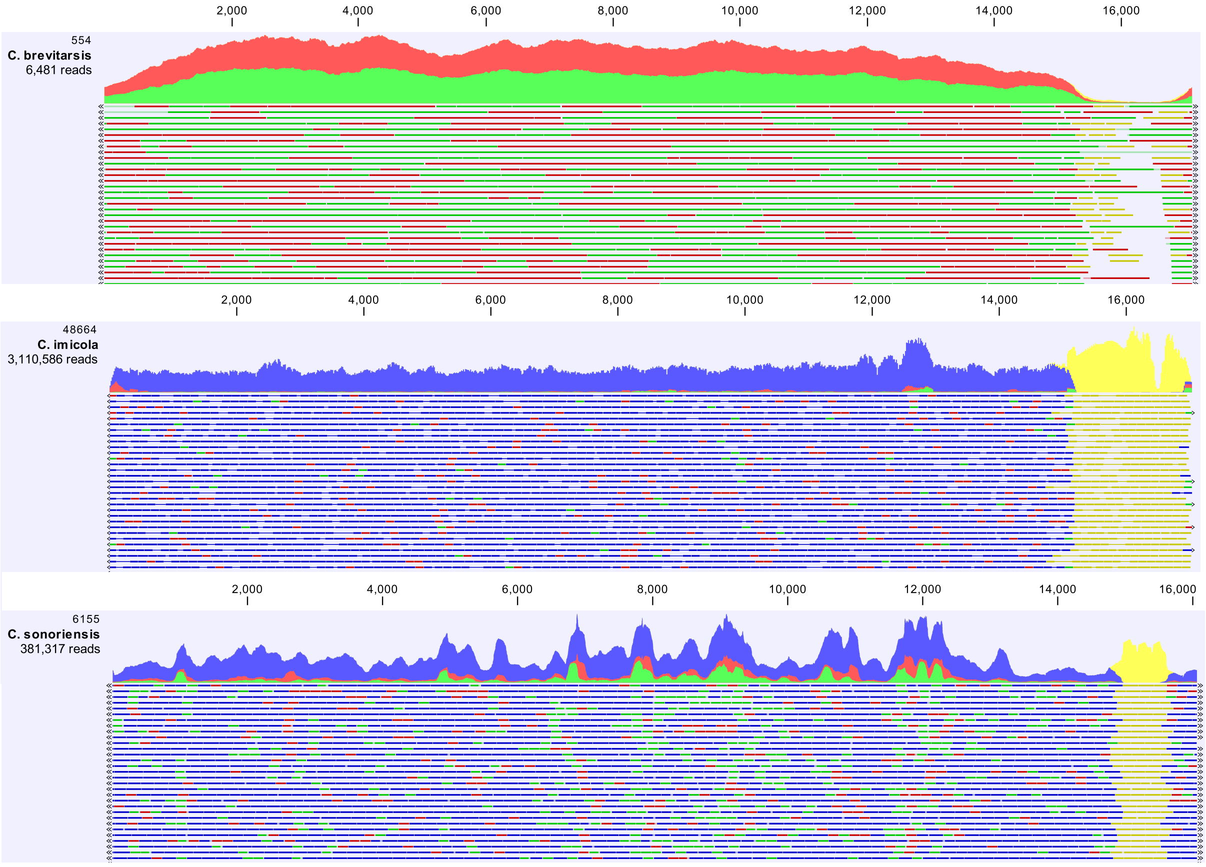

Supplementary Figure S2: Feature annotation maps for mitogenomes of biting midges. The following sequences were used: *C. brevitarsis* (OR730812), *C. imicola* (PQ073138), *C. sonorensis* (BK065013), *C. arakawae* (AB361004), *C. stellifer* (PP873183), *F. makanensis* (MK000395) and *F. pulchrithorax* (OR666457). Protein coding, rRNA genes and control region are shown above each map, tRNAs are indicated below by IUPAC code amino acid transferred. Genes with altered locations relative to *C. brevitarsis* and *C. imicola* are highlighted in blue text.

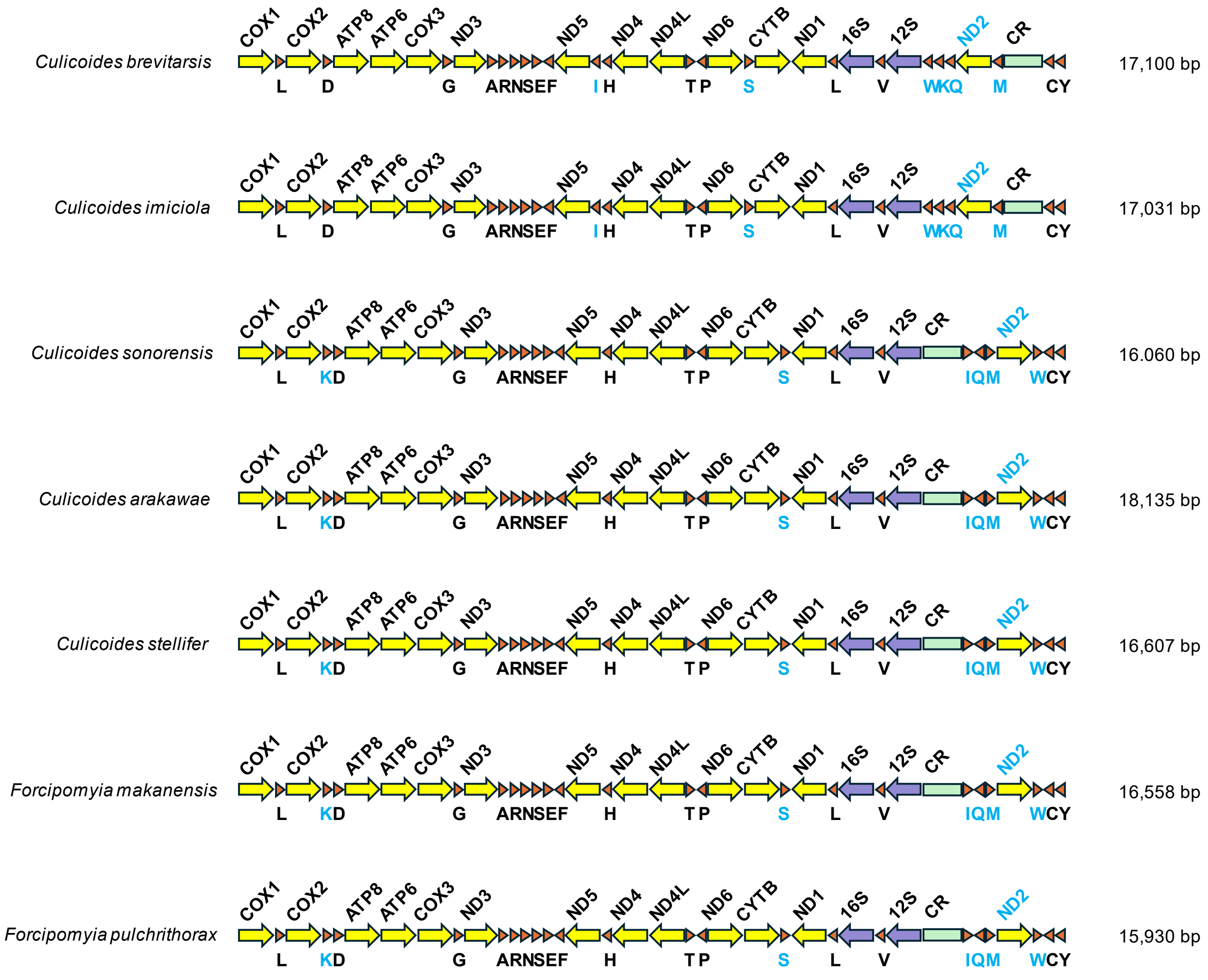

Supplement: supplementary_figs.pdf [file TMDN_A_2447750_SM9582.pdf]
